# Skeletal muscle toxicity associated with tyrosine kinase inhibitor therapy in patients with chronic myeloid leukemia

**DOI:** 10.1038/s41375-019-0443-7

**Published:** 2019-03-14

**Authors:** L. Janssen, S. J. C. M. Frambach, N. A. E. Allard, M. T. E. Hopman, T. J. J. Schirris, N. C. Voermans, R. J. Rodenburg, N. M. A. Blijlevens, S. Timmers

**Affiliations:** 1 Radboud Institute for Health Sciences, Department of Physiology, Radboud University Medical Center, Nijmegen, The Netherlands; 2 Radboud Institute for Health Sciences, Department of Hematology, Radboud University Medical Center, Nijmegen, The Netherlands; 3 Department of Pharmacology and Toxicology, Radboud Institute for Molecular Life Sciences, Nijmegen, The Netherlands; 4 Center for Systems Biology and Bioenergetics, Radboud Center for Mitochondrial Medicine, Radboud University Medical Center, Nijmegen, The Netherlands; 5 Department of Neurology, Donders Institute for Brain, Cognition, and Behaviour, Radboud University Medical Center, Nijmegen, the Netherlands; 6 Radboud Center for Mitochondrial Medicine, Translational Metabolic Laboratory, Department of Pediatrics, Radboud University Medical Center, Nijmegen, The Netherlands; 7 Human and Animal Physiology, Wageningen University, Wageningen, The Netherlands

**Keywords:** Chronic myeloid leukaemia, Translational research

## To the Editor:

Up to 80% of CML patients using tyrosine kinase inhibitors (TKIs) reports muscle complaints [1]. These muscle complaints are strongly related to the presence of fatigue and contributes to both diminished disease control [2] and impaired quality of life [3]. Although the mechanism by which TKIs cause muscle complaints is poorly understood, mitochondrial dysfunction has been suggested to play a pivotal role in TKI-induced cardiac muscle toxicity [4, 5]. We assessed whether TKIs disturb skeletal muscle mitochondrial density and function (cellular level), and if this translates into alterations in muscle contractile function (muscle tissue level) and maximal exercise performance (whole-body level). To gain a better insight into TKI-induced muscle complaints, these outcomes were compared between CML patients with and without muscle complaints. Written informed consent was obtained from the participants prior to study enrollment. This study was approved by the Local Committee on Research Involving Human Subjects of the region Arnhem and Nijmegen, the Netherlands and registered at The Netherlands Trial Registry (NTR6373).

A total of twenty Ph^+^ CP-CML patients on TKI therapy aged ≥ 18 were recruited from the Department of Hematology at the Radboud University Medical Center (Nijmegen, The Netherlands). CML patients were assigned to a group with (CML + MC, *N* = 10) or without (CML - MC, *N* = 10) muscle complaints (MC) on the basis of presence, onset and course of muscle cramps, pain, and/or weakness. This was quantified on a Likert scale from 1 (not at all) to 4 (very much) resulting in significant different scores between CML + MC and CML - MC (median 4.0 (interquartile range (IQR) 3.0–4.0) and 2.5 (IQR 2.0–3.0), respectively; *P* = 0.002). The Brief Fatigue Inventory (BFI) [6] was used to compare the degree of fatigue in CML patients, showing higher fatigue levels in CML + MC when compared to CML - MC (3.58 ± 2.19 and 0.95 ± 1.11, respectively; *P* = 0.005). Ten control participants were matched on group level for age, gender, BMI and physical activity level, assessed by The Short Questionnaire to Assess Health-Enhancing Physical Activity (SQUASH) [7]. Subjects were ineligible if they had hereditary muscle defects, diabetes mellitus, hypo- or hyperthyroidism, severe electrolyte disturbances, or used co-medication known to cause muscle symptoms or have an effect on mitochondrial function (*e.g*. statins, steroids, and metformin). Furthermore, subjects with contra-indications for maximal exercise testing according to the ACC/AHA guidelines [8] and muscle biopsy (e.g. anticoagulant therapy, bleeding disorders) were excluded. The demographic and hematological characteristics shown in Table 1 are not statistically significant different between CML patients and controls and between CML + MC and CML - MC, except for a higher Charlson Comorbidity Index [9] score in CML patients when compared to controls (median 2.0 (IQR 2.0–2.0) and 0.0 (IQR 0.0–0.0), respectively; *P* < 0.001) caused by the presence of CML. All participants completed the study protocol, i.e. a *vastus lateralis* muscle biopsy, electrical *quadriceps femoris* stimulations, and an incremental cycling test.

**Table 1:** Subject and hematological characteristics

Characteristics	CML patients	Controls	*P* value	CML + MC	CML - MC	*P* value
Subject number, *N*	20	10	–	10	10	–
Gender, male/female	14/6	7/3	1.00	7/3	7/3	1.00
Age, years	54 ± 8	58 ± 7	0.25	55 ± 9	54 ± 8	0.86
BMI, kg/m^2^	25.8 ± 4.1	27.5 ± 5.4	0.34	25.8 ± 3.6	25.8 ± 4.8	0.97
Physical activity, METmin/week; median (IQR)	2288 (1545–4982)	2850 (1965–4868)	0.53	2541 (1689–5488)	2070 (1403–4570)	0.63
Smoker, %	0	0	–	0	0	–
Age at Dx, years	46 ± 8	N/A	–	47 ± 7	46 ± 9	0.78
BCR-ABL level, *N* (%)		N/A	–			1.00
No MMR	1 (5)			1 (10)	0 (0)	
MMR or deeper	19 (95%)			9 (90)	10 (100)	
TKI, *N* (%)			–			0.89
Imatinib	10 (50)	N/A		6 (60)	4 (40)	
Dasatinib	5 (25)	N/A		2 (20)	3 (30)	
Nilotinib	2 (10)	N/A		1 (10)	1 (10)	
Bosutinib	2 (10)	N/A		1 (10)	1 (10)	
Ponatinib	1 (5)	N/A		0 (0)	1 (10)	
Duration of current TKI therapy, months; median (IQR)	42.5 (21.0–114.8)	N/A	–	66.5 (19.0–121.5)	38.0 (16.5–73.3)	0.63
Prior TKIs, *N*; median (IQR)	0.5 (0.0–1.0)	N/A	–	0.0 (0.0–1.0)	1.0 (0.0–1.0)	0.53
Charslon Comorbidity Index; median (IQR)	2.0 (2.0–2.0)	0.0 (0.0–0.0)	<0.001	2.0 (2.0–2.0)	2.0 (2.0–2.0)	0.74
Potassium, mmol/l	3.9 ± 0.28	4.0 ± 0.20	0.50	3.9 ± 0.21	4.0 ± 0.34	0.76
Magnesium, mmol/l	0.82 ± 0.06	0.84 ± 0.06	0.49	0.82 ± 0.05	0.83 ± 0.06	0.63
Phosphate, mmol/l	0.82 ± 0.12	0.91 ± 0.17	0.11	0.83 ± 0.10	0.81 ± 0.15	0.75
Calcium, mmol/l	2.29 ± 0.09	2.30 ± 0.06	0.68	2.29 ± 0.09	2.29 ± 0.10	0.91
Albumin, g/l	38.7 ± 2.5	37.3 ± 2.0	0.13	39.1 ± 2.8	38.3 ± 2.2	0.48
TSH, mE/l; median (IQR)	2.1 (1.4–2.6)	2.0 (1.3–2.8)	0.90	2.0 (0.8–2.9)	2.1 (1.5–2.7)	0.61
CK, U/l; median (IQR)	137 (89–236)	120 (109–166)	0.61	169 (103–312)	127 (84–216)	0.90

Values are presented as mean ± SD unless indicated otherwise. There were no significant differences in subject and hematological characteristics between CML patients, except for a higher Charslon Comorbidity Index score in CML patients Also, there were no significant differences between CML patients with and without TKI induced muscle complaints

*MC* muscle complaints, *BMI* body mass index, *MET* metabolic equivalent of task, *IQR* interquartile range, *Dx* diagnosis, *MMR* major molecular response, *TKI* tyrosine kinase inhibitor, *TSH* thyroid-stimulating hormone, *CK* creatine kinase

**Fig. 1:**
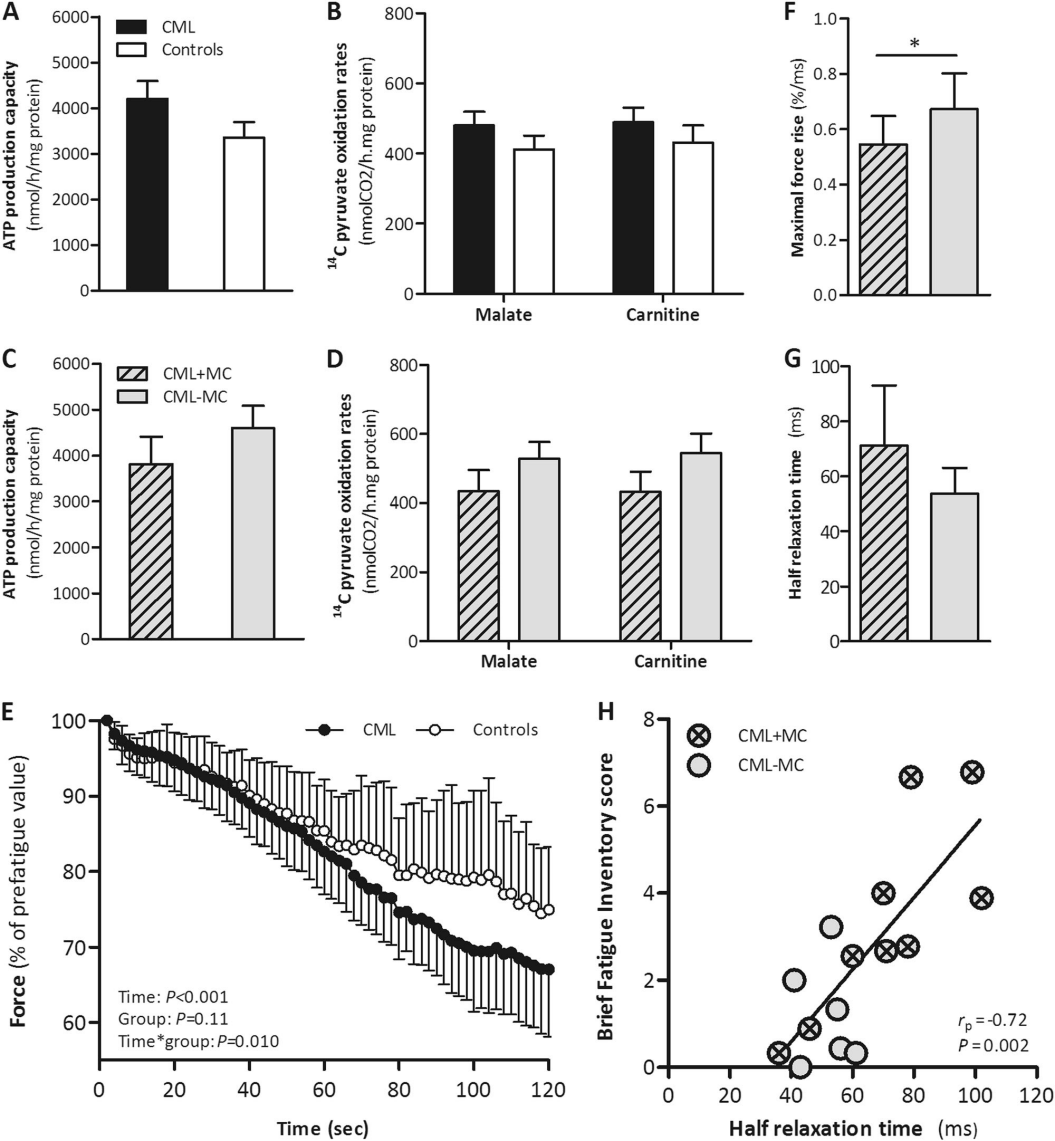
Skeletal muscle mitochondrial function and muscle function parameters measured in CML patients and controls. **a** ATP production rate normalized to muscle protein content and (**b**) [1-^14^C]-pyruvate oxidation rates in the presence of malate and carnitine normalized to muscle protein content measured in mitochondrial fractions from fresh *vastus lateralis* muscle biopsies were not affected by TKI use. There were also no differences in (**c**) ATP production rate and (**d**) [1-^14^C]-pyruvate oxidation rates in the presence of malate and carnitine between CML + MC and CML - MC. **e** Force decline expressed as percentage of the prefatigue value during two minutes repetitive electrical stimulation of the *quadriceps femoris* muscle shows a higher level of muscle fatigue in CML patients compared to controls. Force responses are plotted every second during the complete (120 sec) fatigue protocol. **f** Maximal force rise after two minutes of electrical *quadriceps femoris* stimulation was significantly lower in CML + MC when compared to CML - MC. **g** Q*uadriceps femoris* muscle in CML + MC showed a tendency toward longer relaxation time after two minute repetitive stimulation when compared to CML - MC. **h** Reported fatigue by CML patients (assessed by the Brief Fatigue Inventory) correlates with half relaxation time in *quadriceps femoris* muscle after two minutes repetitive electrical stimulation (*N* = 15). Data are presented as means ± SEM for **a**–**d**; and means ± SD for **e**–**g**. *P value < 0.05 is considered statistically significant

*Vastus lateralis* muscle needle biopsies were performed under local anesthesia in overnight fasted state and processed for mitochondrial measurements according to standard lab techniques as previously published [10]. Citrate synthase activity, a marker for mitochondrial density, was not different between CML patients and controls (195 ± 80 mU/mg protein and 171 ± 30 mU/mg protein, respectively, *P* *=* 0.24) and between CML + MC and CML - MC (*P* = 0.33). Furthermore, mitochondrial function, assessed by ATP production capacity (Fig. 1a, c) and [1-^14^C]-pyruvate oxidation rates in the presence of malate or carnitine (Fig. 1b, d) was not different between groups.

Maximal voluntary muscle strength of the dominant *quadriceps femoris* muscle [11], did neither differ between CML patients and controls (8.3 ± 2.0 N/kg and 7.9 ± 1.8 N/kg, respectively; *P* *=* 0.59), nor between CML + MC and CML-MC (*P* *=* 0.97). Resistance to fatigue was assessed by electrically stimulating the *quadriceps femoris* muscle repetitively at 40% of the MVC using 30 Hz bursts of one-second duration every other second for two minutes [11]. This fatigue protocol resulted in a significantly larger force decline in CML patients as compared to controls (31.8 ± 8.7% and 23.6 ± 7.7%, respectively; *P* = 0.010; Fig. 1e). Although a similar fatigability pattern was observed between CML + MC and CML - MC (force decline 29 ± 9% and 34 ± 9%, respectively; *P* = 0.24), the contractile properties of the *quadriceps femoris* muscle during repeated stimulation were explored in more detail. After two minutes of stimulation CML + MC showed a significantly lower maximal force rise (maximal slope of force increment normalized for peak force) compared to CML - MC (0.54 ± 0.10%/ms and 0.67 ± 0.13%/ms, respectively; *P* = 0.038; Fig. 1f) and a tendency toward longer half relaxation time (time taken for force to decline from 50 to 25% of the peak force; *P* = 0.07; Fig. 1g). The half relaxation time at the end of the fatigue protocol strongly correlated with reported fatigue (*r*_*p*_ = 0.72; *P* = 0.002; Fig. 1h). Since muscle relaxation is dependent upon the activity of sarco/endoplasmic reticulum Ca^2+^-ATPase (SERCA), an enzyme that mediates the re-uptake of calcium into the sarcoplasmic reticulum (SR) of skeletal muscle, SERCA activity was measured in whole-muscle homogenates [11]. However, no significant differences in SERCA activity were observed between CML + MC and CML - MC (98.9 (IQR 79.3–110.7 mU/mg) and 101.5 (IQR 77.0–109.8 mU/mg), respectively; *P* = 0.97).

To measure whole-body fitness levels, all subjects performed an incremental cycle ergometer test (Lode Excalibur; Groningen, the Netherlands) with continuous ECG monitoring [10] to assess peak oxygen uptake (VO_2peak_). VO_2peak_ was 34.6 ± 8.4 ml/kg/min (100 ± 9% predicted) in the CML patients, which was not different from the controls (VO_2peak_ 35.6 ± 7.5 ml/kg/min, 100 ± 9% predicted; *P* = 0.75). In addition, no differences in VO_2peak_ were observed between CML + MC and CML - MC (*P* = 0.38).

Collectively, CML patients on TKI therapy show no signs of skeletal muscle mitochondrial dysfunction. However, *quadriceps femoris* muscle of TKI users fatigues to a larger extent upon repetitive stimulation when compared to controls. Changes in muscle contractile properties are associated with TKI-induced muscle complaints, as CML + MC show a significant lower maximal force rise and a tendency toward a delayed muscle relaxation after two minutes of electrical *quadriceps femoris* stimulations. CML patients did not have impaired maximal exercise performance.

On a cellular level, no effects of TKI therapy on skeletal muscle mitochondrial density and function were found. These results are in line with the only previous clinical case report in which two CML patients, who had to interrupt or reduce therapy with nilotinib because of muscle pain, failed to show disturbances in mitochondrial oxidative enzyme reactions [12]. Intriguingly, in vitro studies in C2C12 myotubes showed no decline in ATP levels upon short-term imatinib incubation of 30 min [13], whereas long-term TKI-incubation of 24 h showed decreased ATP levels overtime [5, 13].

Disturbances in heart mitochondrial function are suggested to occur secondary to activation of a stress response in the endoplasmic reticulum [4]. Perhaps, in skeletal muscle, changes in the function of other cellular organelles also precede mitochondrial disturbances. In support of this hypothesis, CML patients on TKI therapy showed significantly more muscle fatigue than controls, and CML + MC showed delayed *quadriceps femoris* muscle force generation and a trend toward delayed relaxation in fatigued muscle compared to CML - MC. Since muscle fatigability, force generation, and relaxation are largely dependent on Ca^2+^ regelulation by the SR, changes in SR functioning may underlie these findings [14]. In that respect, disturbances in Ca^2+^ homeostasis [15], and SR abnormalities (i.e., dilated SR with membrane whorls) [4] have been found upon imatinib treatment in myocytes, but have never been linked to muscle complaints. Although we found no difference in SERCA activity between CML + MC and CML - MC, muscle half relaxation time after 2-min stimulation correlated positively with the perception of fatigue in CML patients, and may therefore be an important key for understanding the mechanism underlying fatigue in CML.

To the best of our knowledge, maximal exercise capacity has not been assessed before in CML patients or other TKI-users. Compared to controls, CML patients do not have diminished maximal exercise capacity as measured by VO_2peak_ and have similar physical activity levels as controls. VO_2peak_ was also not different between CML + MC and CML - MC, despite higher subjective fatigue levels in CML + MC. These findings fit with the unaltered mitochondrial ATP production capacity, which is an important determinant of VO2_2peak_.

There are several limitations to this study. Due to the exploratory character of the study a relatively large number of measurements were performed in a small sample size. Therefore, results should be cautiously interpreted. Nonetheless, this design made it possible to examine the influence of TKIs on multiple levels (i.e. cellular, muscle tissue and whole body level) which offers broad insight into the effects of TKIs in CML patients. Secondly, participants were only included when they were able to perform all study measurements. Thus patients who were unable to perform exercise testing were excluded. Consequently, extreme cases of TKI-induced skeletal muscle complaints were not included in this study, which may have underestimated the results.

This study provides important information concerning the effects of TKIs on skeletal muscle function and whole body fitness and lays foundation for further studies to elucidate the precise mechanism by which TKI therapy causes muscle complaints and affects muscle function.
